# Factors affecting the extent of resection and neurological outcomes following transopercular resection of insular gliomas

**DOI:** 10.1007/s00701-024-06124-9

**Published:** 2024-06-01

**Authors:** Chandrima Biswas, Prakash M. Shetty, Arpita Sahu, Parthiban Velayutham, Vikas Singh, Kanchi Shah, Aliasgar V. Moiyadi

**Affiliations:** 1 Neurosurgical Services, Department of Surgical Oncology, Tata Memorial Centre, Mumbai, Maharashtra India; 2 Department of Health Sciences, Homi Bhabha National Institute, Mumbai, Maharashtra India; 3 Department of Radiodiagnosis, Tata Memorial Centre, Mumbai, Maharashtra India

**Keywords:** Insular Glioma, Transopercular Resection, Extent of Resection, Neurologic Deficit, MEP, Ischemia

## Abstract

**Background:**

Surgical resection of insular gliomas is a challenge. TO resection is considered more versatile and has lower risk of vascular damage. In this study, we aimed to understand the factors that affect resection rates, ischemic changes and neurological outcomes and studied the utility of IONM in patients who underwent TO resection for IGs.

**Methods:**

Retrospective analysis of 66 patients with IG who underwent TO resection was performed.

**Results:**

Radical resection was possible in 39% patients. Involvement of zone II and the absence of contrast enhancement predicted lower resection rate. Persistent deficit rate was 10.9%. Although dominant lobe tumors increased immediate deficit and fronto-orbital operculum involvement reduced prolonged deficit rate, no tumor related factor showed significant association with persistent deficits. 45% of patients developed a postoperative infarct, 53% of whom developed deficits. Most affected vascular territory was lenticulostriate (39%). MEP changes were observed in 9/57 patients. 67% of stable TcMEPs and 74.5% of stable strip MEPs did not develop any postoperative motor deficits. Long-term deficits were seen in 3 and 6% patients with stable TcMEP and strip MEPs respectively. In contrast, 25% and 50% of patients with reversible strip MEP and Tc MEP changes respectively had persistent motor deficits. DWI changes were clinically more relevant when accompanied by MEP changes intraoperatively, with persistent deficit rates three times greater when MEP changes occurred than when MEPs were stable.

**Conclusion:**

Radical resection can be achieved in large, multizone IGs, with reasonable outcomes using TO approach and multimodal intraoperative strategy with IONM.

**Supplementary Information:**

The online version contains supplementary material available at 10.1007/s00701-024-06124-9.

## Introduction

Insular gliomas (IGs) have fascinated neurosurgeons since the early beginnings of modern microsurgery. They remain the final frontier in glioma surgery. The complex anatomy of the insula, which is buried deep within the opercula, enshrouded in eloquent cortical and subcortical substrates, and crisscrossed with very crucial microvasculature, has made radical resections of IGs challenging. With the advent of microsurgical techniques pioneered by Yasargil and improvements in intraoperative adjuncts, including awake mapping, radical resection with minimal morbidity has become possible. Both trans-sylvian (TS) and trans-opercular (TO) approaches have been described. The TO approach coupled with subpial resection has distinct advantages over the TS approach, especially with regards to minimal direct handling and dissection of the vessels in the sylvian fissure. In addition to direct cortical injury, ischemic injury to major vessels and perforators remains a major contributing factor to postoperative neurological deficits. Indeed, various studies have documented the relative benefits of a TO approach in minimizing such deficits [26, 27]. In this study, we analyzed our experience with the TO approach, aiming to understand the different patient- and tumor-specific factors that affect resection rates, ischemic changes and neurological outcomes, and also studied the utility of intraoperative neurophysiological monitoring.

## Materials and methods

### Patient selection

**Fig. 1:**
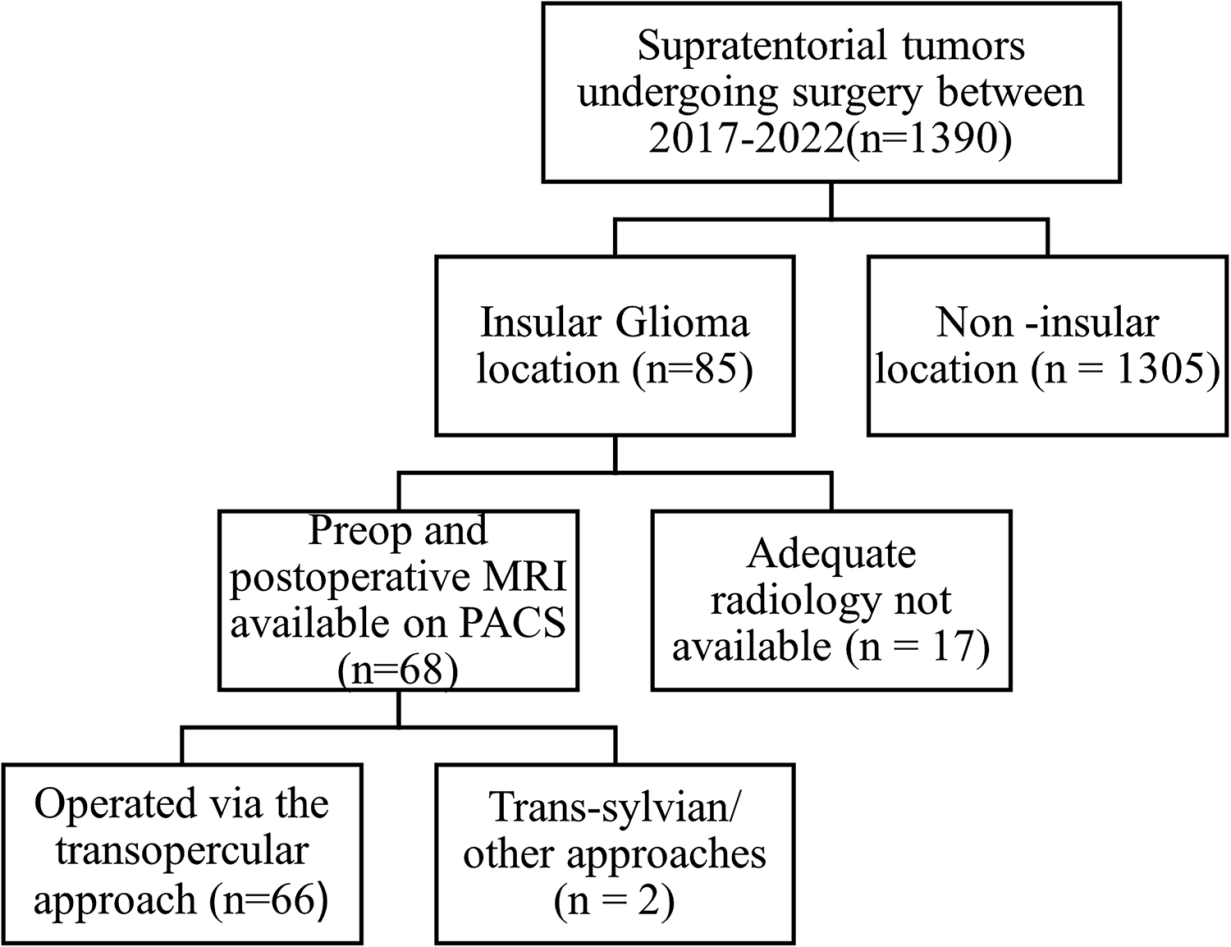
Depiction of the STROBE study flowchart

This was a retrospective review of prospectively collected data. The required authorization (institutional ethics committee approval – IEC no. 3988) was obtained, and informed consent was waived as per institutional stipulations for such studies. The STROBE guidelines (Fig. 1) were followed for the study. All consecutive patients with supratentorial tumors who underwent resection between January 2017 and December 2022 were screened. The criteria for inclusion were gliomas with an insular location, the availability of preoperative and postoperative high-resolution MRI volumetric sequences, and the use of the TO approach. Clinical data were collected from a prospectively maintained neurosurgical database and institutional electronic medical records (EMRs), and radiological data were obtained from the hospital picture archiving and communication system (PACS). Histology was reported as per the 2021 WHO guidelines.

### Surgical technique

As a tertiary referral center, most of our patients with insular gliomas have large or multicompartmental disease precluding resection via the TS route alone. Hence, the TO route is our preferred approach. The anesthesia protocol (asleep/awake) was determined by the type and location of the tumor as well as a presurgical evaluation (including neuropsychological assessment and anesthesia tolerance) of the patients. Generally, we favor awake craniotomy (AC) for lower-grade gliomas (affecting either right or left hemisphere) over glioblastomas. AC was performed using the “asleep-awake-asleep” protocol with patients placed in the lateral position. All patients underwent clinical (when awake) and electrophysiological monitoring (done for both AC and GA). We used a NimEclipse (version 4.2.422; Medtronic Inc., Minneapolis, MA, USA). MN, USA) system. Transcranial electrical stimulation was performed in asleep patients using a multipulse paradigm and suprathreshold stimulation (+ 75 V above threshold), a slight modification from our previously reported protocol [22]. This, in our experience, reduces the variability of MEPs recorded. We also performed direct cortical stimulation (DCS) using a strip electrode whenever possible while the patients were asleep or awake, as well as subcortical suction monopolar motor mapping as we have previously described [28]. A standard wide frontotemporal craniotomy was performed. Pre-resection ultrasound scans were performed in most patients before and/or after dural opening with/without integration with a navigation system (2D/3D US scan). After opening the dura, DCS was used to map the motor and language areas (when performed under awake conditions) using standard language mapping paradigms and bipolar stimulation (60 Hz). Cortical strip electrodes were placed for MEP recordings whenever possible. Following the identification of eloquent areas, cortical incisions were made. The site and number of these cortical windows were determined by the location of the eloquent areas and the extent of the tumor. Thus, this varied on a case-by-case basis. Subsequently, the frontal and temporal opercula were resected. This was followed by subpial resection of the tumor in the insula guided by ultrasound imaging and/or navigation along with continuous clinical/electrophysiological monitoring. Subcortical stimulation (both bipolar 60 Hz with appropriate motor, language and cognitive tests in awake patients and continuous monopolar suction stimulation in asleep and awake patients) was used. Following resection, a final ultrasound scan with/without navigation was performed to assess the extent of resection.

### Warning criteria for MEP changes

Various warning criteria have been described for MEP monitoring in supratentorial tumors, especially IG [1, 14, 18, 23] but there are no definite guidelines. We used the criteria mentioned in Table 1. Changes were categorized by severity (deterioration or loss) as well as reversibility.

**Table 1:** Warning Criteria for MEP changes

Warning Criteria	Strip MEP (threshold stimulus)	TES MEP (suprathreshold stimulus)
Stable (Range of stability)	MEP amplitude changes < 30% from baseline	MEP amplitude changes < 50% from baseline
Deterioration	Amplitude drops below range of stability	
1. Reversible	Recovers to range of stability (at same threshold + 2 mA maximum)	Recovers to range of stability (at same threshold + 100 V maximum)
2. Irreversible	Does not recover (or requires much higher stimulus)	Does not recover (or requires much higher stimulus)
Loss	Complete disappearance of the MEP	
1. Reversible	Recovers to range of stability (at same threshold + 2 mA)	Recovers to range of stability (at same threshold + 100 V)
2. Irreversible	Does not recover (or requires much higher stimulus) to that range	Does not recover (or requires much higher stimulus) to that range

### Imaging analysis

Preoperative and postoperative MR images were reviewed by one of the trained neurosurgeons (CB). All postoperative scans were performed within 48 h. Tumors were categorized radiologically using the Yasargil and Berger Sanai classifications [30, 35]. The EOR for each patient was measured volumetrically by a neurosurgeon (CB) and confirmed by a senior neuroradiologist (AS). Tumor volume (cm^3^) was calculated on MRI (T2-weighted sequence for non-enhancing tumors and volume of contrast-enhancing tissue on gadolinium-contrast T1-weighted images for enhancing lesions) in DICOM format, followed by volumetric quantification with insight registration and segmentation toolkit software (ITK-SNAP version 4.0.0, http://www.itksnap.org). The EOR was calculated using the following formula: [(preoperative tumor volume—postoperative tumor volume)/preoperative tumor volume × 100%]. Resection was grouped into radical resection (EOR > 90%) and nonradical resection (EOR < 90%). Postoperative DWI was reviewed to check for significant postoperative ischemia, and the results were classified according to our previously reported schema [20]. Significant ischemia was classified as arterial or venous (thick peri-resection cavity DWI changes). Arterial ischemia was further classified based on the territory involved (lenticulostriate, MCA, and so on).

### Neurological and neuropsychological assessment

The appearance of any new neurological worsening after surgery for 48 h was defined as a deficit, and its evolution over time was studied. Deficits that occurred immediately following surgery were termed “immediate” deficits, and those that occurred between 24–48 h were considered “delayed”. The deficit was “transient” in nature if it resolved by the time the patient was discharged and “prolonged” if it resolved within 3–6 months. Any deficit present at 3–6 months was termed “persistent”.

A detailed neuropsychological assessment was conducted by a trained neuropsychologist (KS) prior to surgery and during the follow-up period prior to the start of adjuvant therapy wherever possible as part of routine practice. Specific neurocognitive tests pertaining to the domains of attention/executive functions, memory, language, visuospatial function and visuomotor function were conducted as per a protocol we previously published [21].

### Statistical analyses

Descriptive analysis was performed for all the variables, and categorical data are represented as percentages, while continuous data are presented as the mean/median as appropriate. MEP changes were correlated with EOR, DWI changes and neurological outcomes using the chi-square test or Fisher’s exact test (if more than 80% of the values were less than 5). Since MEPs can assess only motor pathway integrity, only motor deficits were considered in their assessment and correlation. DWI changes were also correlated with EOR and neurological outcomes. To determine the factors affecting EOR and neurological outcomes (immediate, prolonged and persistent deficits), univariate analyses were carried out using the chi-square test for comparing categorical variables and Student’s t test for comparing the means of continuous variables, as appropriate. Variables with p < 0.2 were entered into a multivariate model (stepwise logistic regression). The final models retained only the variables significant at the p < 0.05 level. Analyses were performed using IBM SPSS software (version 25, IBM Corp.).

## Results

### Patient characteristics

Sixty-six patients underwent TO resection during the study period from 2017–2022. Figure 1 shows the outline of the included study cases. The cohort consisted predominantly of males (71.2%), with a mean age of 39 years. Most (71.2%) presented with seizures, with a mean duration of symptoms of approximately 10 months. Table 2 shows the detailed clinicopathological features of our patient population. Most of the tumors (77.3%) were lower grade (grades 2 and 3) gliomas, and astrocytic tumors were more common than oligodendroglial tumors. All the oligodendrogliomas (1p19q codeleted) were histologically grade 3. Of the grade 4 tumors observed in 15 (22.7%) patients, 13 had glioblastomas, and 2 had Grade 4 IDH mutant astrocytomas. Similar to our previous observation [21] severe baseline neurocognitive deficits were seen in 90% of the patients and the most commonly affected domains were attention and memory.

**Table 2:** Demographic, clinical and pathological characteristics of the patient cohort

Patient Characteristics	Patients number(%)
Age (mean ± SD)	39 ± 11.9 years
Gender	
1. Males	47(71.2%)
2. Females	19(28.8%)
Presenting symptoms:	
1. Seizures	47(71.2%)
2. Neurological deficit	15(22.7%)
3. Raised ICP	7(10.6%)
Duration of presenting symptoms (mean ± SD)	9.77 ± 16.4 months
KPS	
1. < 70	2 (3.0%)
2. 70–80	21(31.9%)
3. > 80	43(65.1%)
Co-morbidities	7(10.6%)
Prior treatment received	14(21.2%)
1. Surgery alone	10(71.4%)
2. Surgery with adjuvant RT	2(14.3%)
3. Surgery with adjuvant RT and chemotherapy	2(14.3%)
Baseline Neuropsychological assessment done	41(62.1%)
1. Severely affected overall neurocognitive function	37(90.2%)
2. Severely affected Memory	23(56.1%)
3. Severely affected Attention	35(85.4%)
4. Severely affected Language	10(24.4%)
5. Severely affected Visuospatial	18(43.9%)
6. Severely affected Visuomotor	16(39%)
Tumor Grade	
1. Lower grade (WHO grade 2 or 3)	51(77.3%)
2. High Grade (Grade 4 astrocytoma/GBM)	15(22.7%)
Tumor lineage/ type	
1. Astrocytic	49 (74.2%)
2. Oligodendroglial	17(25.8%)

### Radiological characteristics

**Fig. 2:**
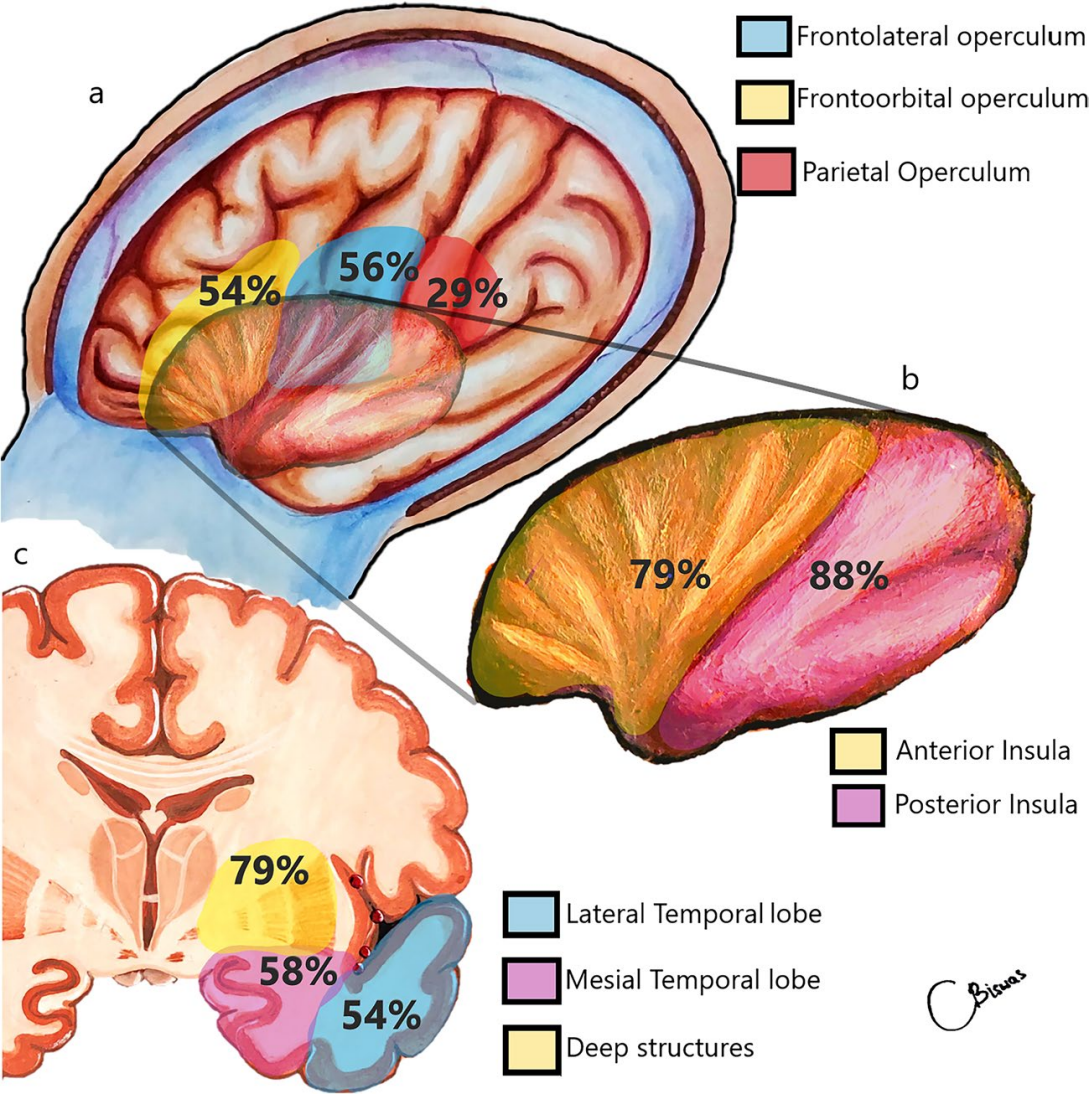
Extent of involvement of insular gliomas within and beyond the insula. **a** Exposed brain after wide fronto-temporal craniotomy with impression of insular lobe showing percentages of involvement of individual operculum, **b** Percentages of involvement of different parts of insular lobe, **c** Coronal section through the Foramen of Monroe showing involvement of parts of temporal lobe and deep structures

Tumors were equally distributed across both the left and right sides, with a mean tumor volume of 92.6 ± 47 cm^3^. Table 3 shows the radiological characteristics of the tumors. The majority of the tumors (82%) had a surfacing component, with opercular involvement observed in 72.4%. Figure 2 shows the extent of tumor involvement. Deep structures, including the gangliocapsular region, basal forebrain and anterior perforated substance (APS), were involved in 79% of the patients. A total of 58% of the tumors extended to the medial temporal lobe (Yasargil type 5B), whereas pure insular (type 3A) tumors were observed in only 6% of the patients.

**Table 3:** Detailed radiological characteristics and extent of the involvement by tumor

Radiological Characteristic	Groups studied	Number of patients (%)
Side	Dominant	34(51.5%)
	Non dominant	32(48.5%)
Localization	Diffuse	19(28.8%)
	Moderately localized	30(45.5%)
	Well localized	17(25.8%)
Enhancement pattern	Non enhancing	31(46.9%)
	Enhancing	35(53%)
Yasargil classification	Group 3A	4(6%)
	Group 3B	7(10.6%)
	Group 5A	17(25.7%)
	Group 5B	38(57.6%)
Berger Sanai Zones involved *	Zone I	43(65.2%)
	Zone II	42(63.6%)
	Zone III	48(72.7%)
	Zone IV	49(74.2%)
Number of Zones involved	1	6(9.1%)
	2	29(43.9%)
	3	6(9.1%)
	4 (Giant)	25(37.9%)

### Intraoperative details

Although an equal number (33 each) of patients underwent surgery under awake conditions and GA, AC was preferred for dominant lobe tumors (64%). In the AC group, motor function was monitored clinically as well as electrophysiologically (cortical strip MEPs) in most cases, whereas language and other neurocognitive functions (attention, task-switching, semantic association) were assessed and monitored meticulously by a neuropsychologist (KS) using our protocol. Ultrasound was used in 64 (97%) patients, whereas navigation was used in 52 (79%) patients. Four patients underwent 5’ALA-guided resection (all grade 4 tumors). Overall, electrophysiological monitoring was used in 57 (86%) patients; transcranial MEPs (TcMEPs) were monitored in 33 patients (all GA patients), and strip MEPs (alone or along with TcMEPs) were monitored in 56 patients. Subcortical dynamic monitoring with a suction monopolar device was performed in 30 (46%) patients, and the lowest motor threshold reached during resection ranged from 4–15 mA, with a mean of 9.5 mA. The intraoperative neuromonitoring findings and changes experienced are summarized in Table 4. Most patients had stable MEPs. Reversible MEP changes occurred at 2/33 Tc and 8/56 strip MEPs. Only 1 patient experienced irreversible loss of both TcMEPs and strip MEPs.

**Table 4:** Details of electrophysiological modalities used for motor monitoring and respective changes seen

Modality	MEP changes	Number of patients (%)
Transcranial MEPs (Recorded in 33, 50%)	Stable	30(90.9)
	Reversible loss	2(6.06)
	Irreversible loss	1(3.03)
Strip MEPs (recorded in 56, 84.8%)	Stable	47(83.9)
	Reversible deterioration	8(14.28)
	Irreversible loss	1(1.78)
Subcortical monopolar mapping (recorded in 30, 45.5%)	Mean lowest motor threshold of resection	9.5 mA
	Range of lowest motor threshold reached during resection	4–15 mA

### Extent of resection

The mean EOR calculated volumetrically was 81.2% ± 18.7%, with a mean residue volume of 19 cm^3^ ± 22.4 cm^3^. A total of 26 (39%) patients underwent radical resection (complete resection in 18.2%, near total resection in 21.2%).

### Neurological outcomes

During the postoperative period, 30 patients developed new neurological deficits. Immediate postoperative worsening was observed in 29 patients, and one patient developed a delayed deficit due to an operative site hematoma associated with an MCA pseudoaneurysm for which he underwent re-exploration and subsequently developed a left-sided MCA territory infarct. Of the 30 patients who experienced postoperative deterioration, 16 experienced transient deterioration, which improved by the time they were discharged (transient deficit rate of 24.2%).

**Fig. 3:**
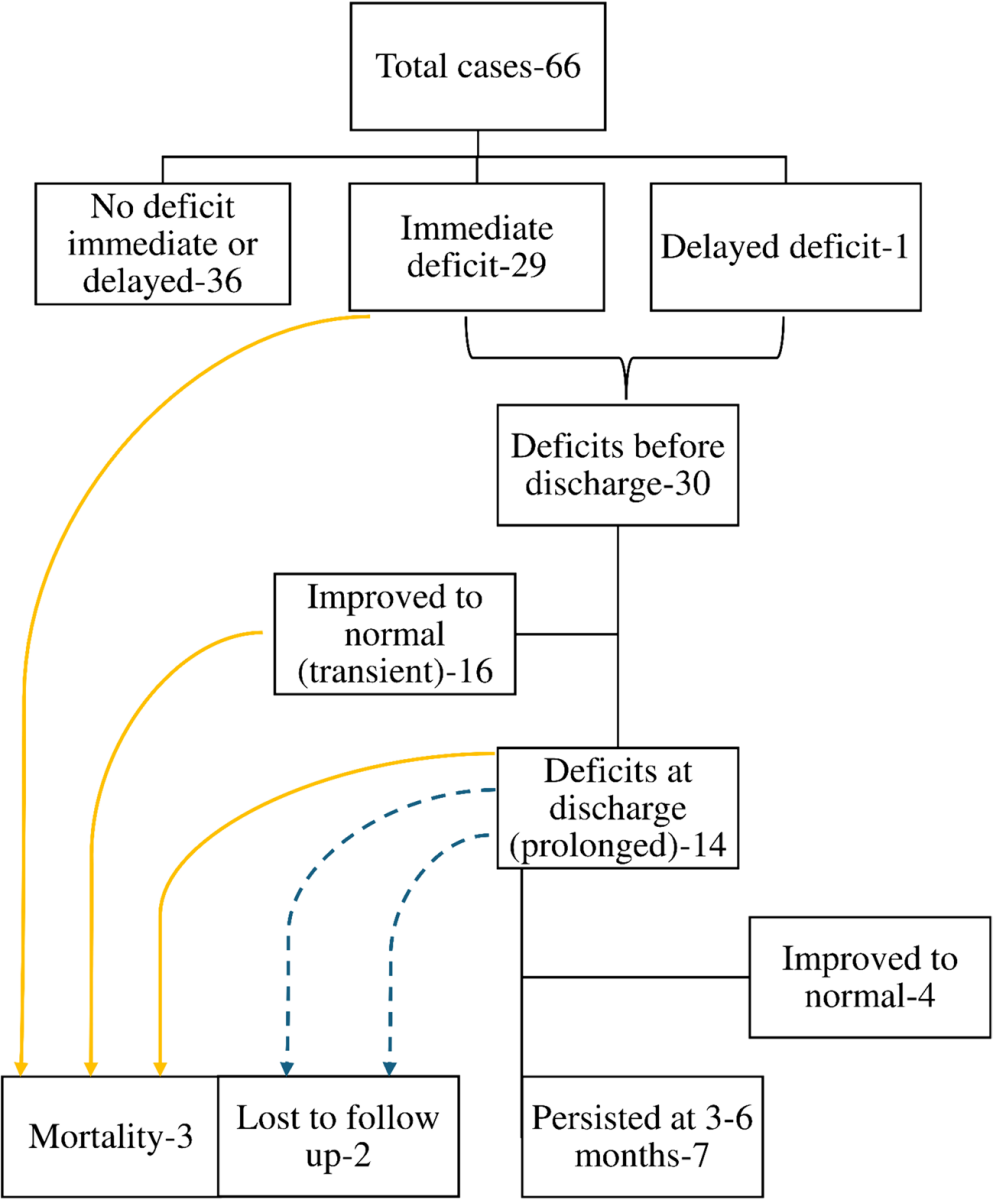
Evolution of deficit

Fourteen (21.8%) patients experienced prolonged deficit at 3–6 months, with one case of perioperative mortality. This patient had a left-sided giant insular glioma and developed irreversible loss of both Tc and strip MEPs intraoperatively. Postoperative MRI revealed a thick perilesional venous infarct. He had a stormy course and eventually succumbed to multiple secondary complications. Two patients were lost to follow-up, and two other patients died from complications unrelated to the surgical intervention. Persistent deficits at 6 months were observed in 7 (10.9%) patients. Figure 3 shows the temporal pattern of the evolution of these deficits.

The radicality of resection did not affect the occurrence of postoperative deficits. The immediate, prolonged, and persistent deficit rates were 50%, 20% and 12%, respectively, for the radical resection group and 42.5%, 23% and 10.2%, respectively, for the nonradical group.

### Postoperative DWI changes

**Fig. 4:**
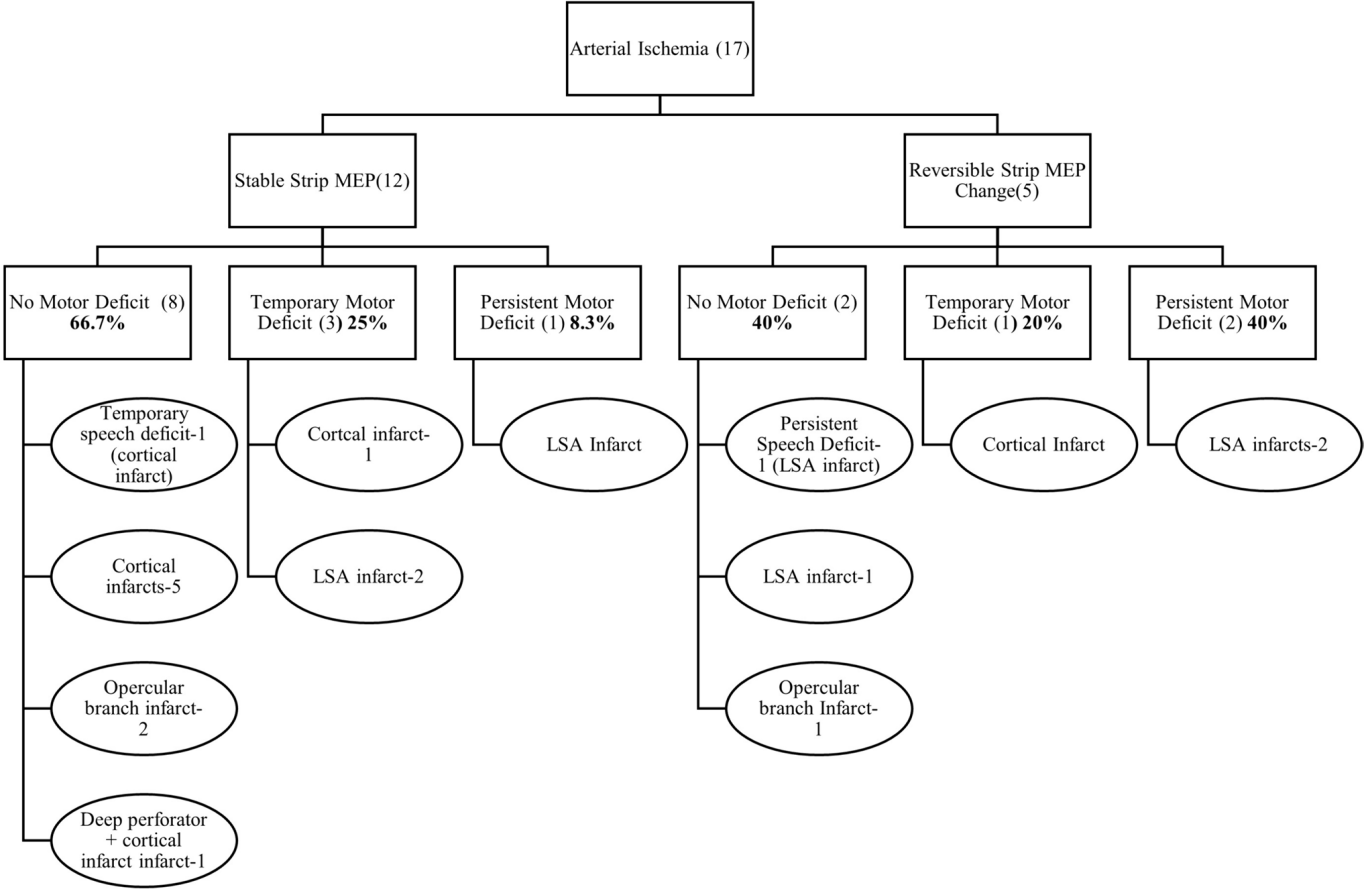
Clinical and Electrophysiological manifestation of Arterial Ischemia and the corresponding arterial territories involved

Thirty patients (46%) had radiologically significant DWI changes on immediate postoperative MRI. Among these patients, one patient had both an arterial (M4 cortical branch) and a thick peri-resection venous infarct. Arterial ischemia was observed in 17 patients, of which a lenticulostriate artery (LSA) infarct was observed in 7 (39%), an opercular (M2-M3) segment infarct was detected in 4 (22%), and cortical terminal branch (M4) infarcts were detected in 39% (4 in frontal, 2 in temporal and 1 in parietal cortical branches). One patient had both cortical and deep perforator infarcts on postoperative MRI. Figure 4 shows the clinical and intraoperative electrophysiological manifestations of patients with arterial ischemia. Most infarcts associated with cortical or opercular branches of the MCA were ‘non’ significant (resulting in no/temporary deficit), whereas LSA infarcts were strongly predictive of a persistent deficit.

### MEP changes and correlation with neurological outcome, EOR and DWI changes

A significant proportion of patients with stable strip and Tc MEPs had no motor deficits (66.7% of Tc MEPs and 74.5% of strip MEPs). Most motor deficits that occurred were temporary (Table 5). However, 3 out of 6 patients with persistent motor deficits did not have any corresponding change in the MEP, possibly suggesting the onset of a delayed ischemic event evading detection by the MEP change criteria we used or the involvement of nonprimary motor substrates that could not be monitored using the MEPs. Nine patients experienced MEP changes during surgery. One patient had irreversible loss in Tc and strip MEPs, two other patients had reversible loss in Tc MEPs and reversible deterioration in strip MEPs, and six patients had reversible deterioration in strip MEPs alone. One-quarter of patients with reversible strip MEP changes and half of patients with reversible Tc MEP changes had persistent motor deficits, whereas one patient with irreversible MEP changes (both Tc and strip) developed hemiplegia. This patient had incomplete resection, although overall, the EOR was not affected by MEP changes (Table 5).

**Table 5:** Relationship between MEP changes, neurological outcomes and EOR

Correlation of MEP Changes and EOR	Tc MEP changes			Strip MEP changes		
	Stable (*N* = 30) *n*(%)	Reversible loss (*N* = 2) *n*(%)	Irreversible loss (*N* = 1) *n*(%)	Stable (*N* = 47) *n*(%)	Reversible deterioration (*N* = 8) *n*(%)	Irreversible loss (*N* = 1) *n*(%)
> 90% EOR (Radical resection)	13(43.3%)	2(100%)	0	15(31.9%)	5(62.5%)	0
	*P* = 0.2			*P* = 0.2		
Correlation of MEP Changes and Deficit	Tc MEP changes			Strip MEP changes		
	Stable (*N* = 30) *n*(%)	Reversible loss (*N* = 2) *n*(%)	Irreversible loss (*N* = 1) *n*(%)	Stable (*N* = 47) *n*(%)	Reversible deterioration (*N* = 8) *n*(%)	Irreversible loss (*N* = 1) *n*(%)
No deficit	20(66.7%)	0	0	35(74.5%)	4 (50%)	0
Transient motor deficit	6(20%)	1(50%)	0	5(10.6%)	2(25%)	0
Prolonged motor deficit	3(10%)	0	0	4(8.5%)	0	0
Persistent motor deficit	1(3.3%)	1(50%)	1(100)	3(6.4%)	2(25%)	1(100)

Postoperative DWI changes did not necessarily indicate postoperative deficits, as shown in Table 6. When analyzed for the type of infarct, arterial ischemia was more clinically relevant, leading to significantly more long-term deficits (both speech and motor), whereas venous ischemia did not always cause clinically relevant deficits (except for the patient with irreversible MEP loss). Conversely, not all patients with postoperative deficits had significant DWI changes. This highlights the role of other mechanisms in causing postoperative deficits, such as direct cortical or subcortical injury.

**Table 6:** Correlation between diffusion changes on MRI and neurological deficits

	Arterial Infarct (N = 17)	Venous infarct (N = 14)	Any infarct (N = 30)
	*n*(%)	*n*(%)	*n*(%)
Immediate deficit Motor Speech Both	9(52.9) *p* = 0.57	8(57.1) *p* = 0.37	16(53.3) *p* = 0.32
	5(29.4) 2(11.8) 2(11.8)	3(21.4) 2(14.3) 3(21.4)	7(23.3) 4(13.3) 5(16.7)
Prolonged deficit Motor Speech Both	**7(41.2) *p* = 0.03**	3(21.4) *p* = 1	9(30) *p* = 0.13
	4(23.5) 2(11.8) 1(6)	1(7.1) 0(0) 2(14.3)	4(13.3) 2(6.7) 3(10)
Persistent deficit Motor Speech Both	**4(23.5) *p* = 0.05**	2(14.3) *p* = 0.82	**6(20) *p* = 0.02**
	3(17.6) 1(6) 0(0)	1(7.1) 0(0) 1(7.1)	4(13.3) 1(3.4) 1(3.4)

**Fig. 5:**
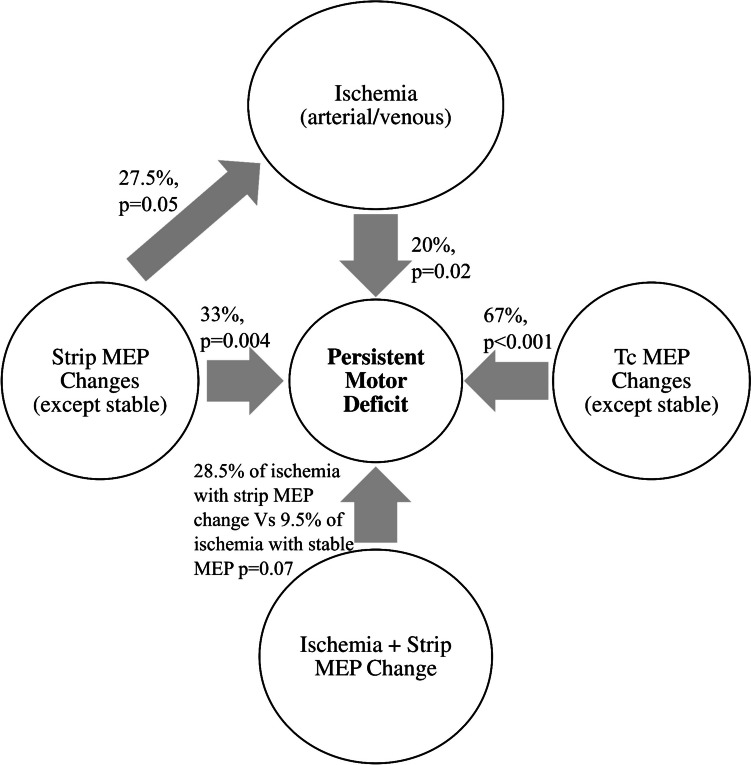
Association (p values) between DWI Changes, MEP changes and Persistent deficit

Furthermore, DWI changes were clinically more relevant when accompanied by MEP changes intraoperatively, with persistent deficit rates three times greater (28.5% vs 9.5%) when MEP changes did occur than when MEPs were stable, regardless of whether the ischemia was venous or arterial (Fig. 5).

### Factors affecting EOR

**Fig. 6:**
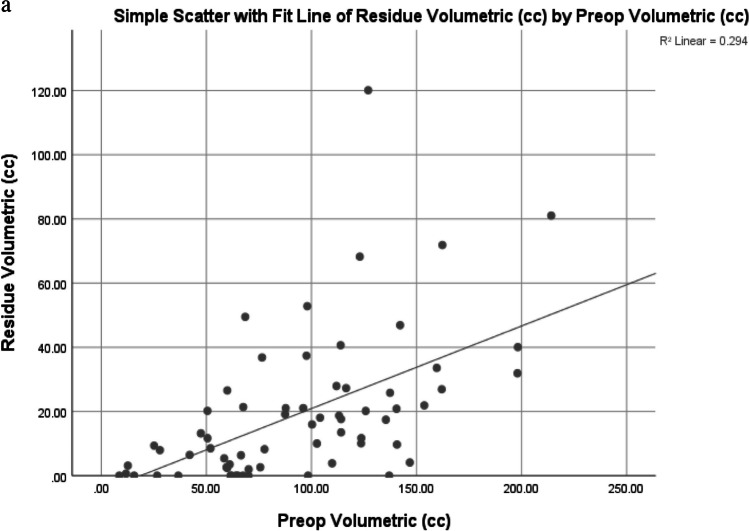

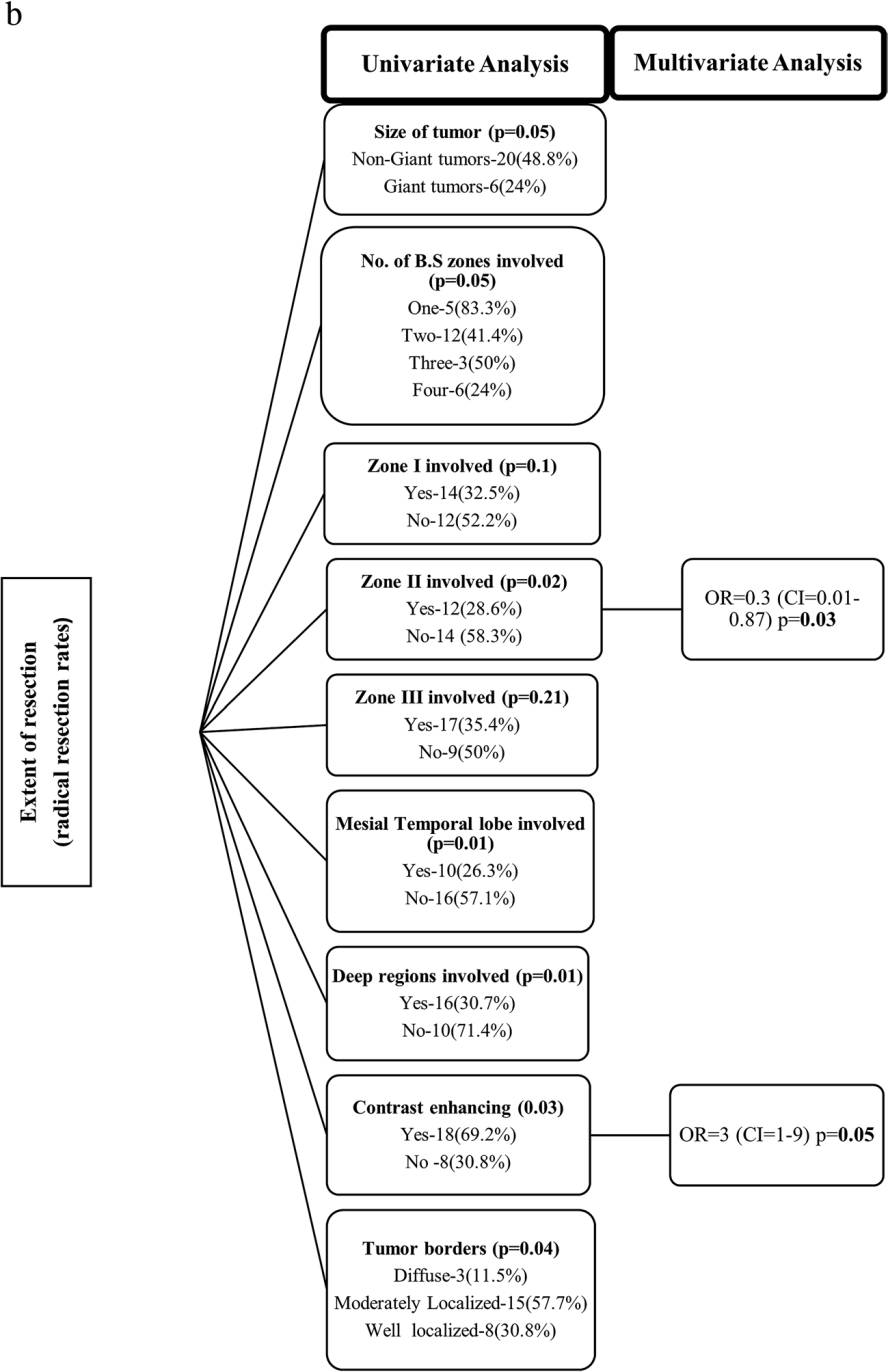
Analysis of factors affecting EOR: **a** Correlation between tumor volume and postoperative residue, **b** Univariate and Multivariate analysis result of relevant variables

According to the univariate analysis, the preoperative tumor volume correlated with the postoperative residual tumor volume (r = 0.54, *p* < 0.001), and giant tumors were less likely to undergo radical resection (although both giant and non-giant tumors had a similar mean EOR of 81%). Tumors involving zone II, the mesial temporal lobe and deep/subcortical eloquent areas were also less likely to undergo radical resection, as were non-enhancing tumors and those with diffuse/ill-defined borders (Fig. 6). There was no difference in the EORs between the awake and GA groups. Multivariate analysis revealed that the involvement of zone II and nonenhancement of the tumor were independent predictors of nonradical resection rates.

### Factors affecting neurological outcomes

**Fig. 7:**
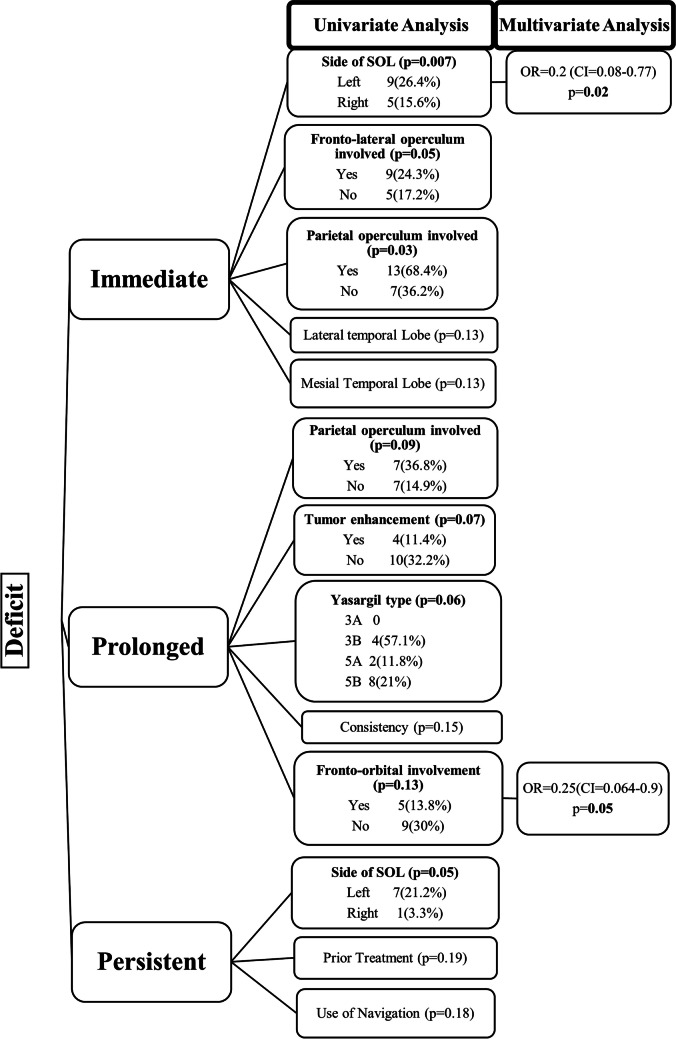
Analysis of factors affecting neurological outcomes

Univariate analysis revealed that dominant lobe tumors contributed significantly to immediate (Fig. 7) and persistent deficits; the involvement of the fronto-lateral and parietal operculum increased the immediate deficit rate, whereas fronto-orbital operculum involvement decreased the prolonged deficit rate. Multivariate analysis revealed that involvement of the dominant lobe was an independent risk factor for immediate deficit (motor and speech), and involvement of the fronto-orbital operculum reduced the likelihood of prolonged deficit (suggesting by corollary, the relative significance of fronto-lateral, parietal, and temporal opercular involvement in producing these deficits). However, in case of persistent deficit none of these factors reached statistical significance. Details of the analysis of all the factors affecting EOR and neurologic outcome have been included in the supplementary material (SM Tables 3, 4).

Patients who underwent AC had rates of postoperative deficit similar to those of patients who underwent monitoring under GA. Further analysis of these motor and speech deficits revealed that AC was associated with significantly fewer transient motor deficits (3%) than GA (21.2%). However, persistent motor deficit rates were similar, and even though a significant proportion of patients who underwent AC had dominant lobe tumors (64% of AC patients vs 39% of GA patients), the speech deficit rates were comparable to those in the GA group (SM Table 2).

## Discussion

Surgery for IGs has remained challenging. Earlier series reporting treatment of IGs [15, 31] were selective in the kind of tumors that underwent radical resection, preferring to resect those patients who were expected to have a better overall prognosis. Patients with poor Karnofsky Performance Scale (KPS), radiological features of high-grade tumors and more extensive Yasargil type 5 tumors generally underwent biopsy or suboptimal resection. With a better understanding of the insula and its functional connectivity and with technical and technological advancements, subsequent series [30, 32] have expanded their patient selection for aggressive resection and have demonstrated that the EOR is an important predictor of overall and progression-free survival.

Our series adds to the body of evidence, especially for large and giant tumors, which are quite typical of our patient profile. Our study is also unique in that in addition to preoperative characteristics, we studied in detail the interactions between MEP changes, ischemia type, EOR and morbidity.

### Radical resections in insular gliomas

Improving EOR remains the primary goal of surgical strategies for IGs due to the prognostic benefit it confers. Radical resection of > 90% has been reported in the literature to range from 12.5% to 74.6%, as shown in Table 7, and our radical resection rate of 39.4% is comparable. Given the complexity of this anatomical region, many factors influence the EOR, and it is very difficult to compare outcomes across series. Tumor location and extent of involvement are major determinants. Posteriorly located insular gliomas (Zone II) are particularly difficult to approach trans-cortically (or even trans-sylvian) by virtue of their proximity to the overlying eloquent frontal and temporal opercula housing the important language and motor areas and the subcortical white fibers at depth. The close approximation and difficulty in splitting the posterior aspect of the sylvian fissure also make trans-sylvian access difficult. Additionally, the posterior insula itself is involved in speech [24]. As a result, this makes zone II tumors the least amenable to GTR, as seen in our series (28.6% radical resection rate) and described in various other series [11, 13, 30]. The higher (35.4%) radical resection rate for Zone III tumors could be attributed to the appropriate use of motor and language mapping. In contrast, anteriorly located tumors involving zones I and IV are considered relatively favorable for radical resection, as seen in studies [9, 11, 16]. We found that although the involvement of Zone II had the lowest radical resection rate, this difference was not large compared to the involvement of other zones. This was because many of the tumors involved multiple zones. Thus, a tumor involving zone I could have undergone nonradical resection because the tumor also involved zone II. Additionally, the involvement of deep eloquent substrates could have led to subtotal resections in Zone I and IV tumors (wherein the tumor in Zone I/IV itself may have been resected). In fact, patients with APS are more likely to have Zone I/IV involvement due to contiguity. This highlights a potential drawback of using the Berger Sanai classification, and specifying the involvement of subcortical eloquent substrates is also important when discussing EOR outcomes in IGs. Moreover, giant IGs were associated with a lower radical resection rate in the univariate analysis, which was also observed in other studies [9, 30].

**Table 7:** Literature review including relevant information linked to neurological outcome and EOR

Study	Year	Sample size	Resection Route	Volu metric	Preop Tumor Volume- (cc)	Radical resection rate (%)	EOR (%)	Periop Mortality(%)	Immed ND(%)	Persistent ND(%)	Awake Cran(%)	Dominant Lobe(%)	HGG
Yaşargil and Reeves, 1992 [34]	1992	240	TS	No	*	*	*	0	*	5	0	53	43.5
Lang et al*.*, 2001[15]	2001	22	TO + TS	Yes	46.5^a^	45.5	86^a^	0	36.4	9.1	22.7	59.1	50
Simon et al*.*, 2009[31]	2009	94	TO + TS	No	*	42	*	3	*	20	0	40	20
Duffau, Moritz-Gasser and Gatignol, 2009 [6]	2009	24	TO	No	59^a^	12.5	*	0	50	0	100	100	0
Duffau, 2009[5]	2009	51	TO + TS	No	65^a^	16	*	0	58.8	3.9	31.4	27.4	0
Sanai, Polley and Berger, 2010[30]	2010	104	TO	Yes	*	23	84.5^a^	0	14.4	5.7	56.5	55.8	8.7
Skrap et al*.*, 2012[32]	2012	66	TO + TS	Yes	108^b^	33	80^a^	0	33.4	3	66	66.7	0
Kawaguchi et al*.*, 2014[13]	2014	83	*	Yes	*	44.6	*	0	26.5	16.9	6	55.4	37.3
Hervey-Jumper et al*.*, 2016[11]	2016	114	TO	Yes	48.5^a^	39.5	85^a^	0	26.4	3.2	45	52.7	11.6
Eseonu et al*.*, 2017[7]	2017	74	TO	Yes	43.6^a^	54	91.7^a^	0	19	2.7	39.2	58.1	44.6
Przybylowski et al*.*, 2020[27]	2018	100	TO + TS	Yes	62.4^a^	*	88.6^b^ (TO)	0	18	10	12	60	68
Mandonnet, 2019[19]	2019	12	TO	Yes	68.5^a^	50	94^a^	0	8.3	0	83.3	58.3	0
Lu et al*.*, 2019[17]	2019	890	*	Yes (8/19 studies)	69^b^	57	87^b^	*	22	6	34	52	44
Di Carlo et al*.*, 2020[4]	2019	227	TO + TS	Yes (6/8 studies)	75.4^b^	27.4	*	*	33.6	10.6	30.7	60	28
Hameed et al*.*, 2019[9]	2019	255	TO	Yes	70.4^a^	67.5	97.95^a^ 91.6^b^	0.4	19.2	15.7	*	56.8	31.8
Li et al*.*, 2020[16]	2020	253	TO	Yes	78.3^a^	69.6	*	0	20.2	3.2	0	47	11.9
Rossi et al*.*, 2021[29]	2021	95	TO	Yes	76.3^b^ 73.5^a^	73.7	92.3^b^ 99^a^	0	54.7	5.2	73.6	63.1	20
Panigrahi et al*.*, 2021[26]	2021	61	TO + TS	No	*	62.3	*	9.8	47.5	11.5	0	42.7	19.6
Pallud et al*.*, 2021[25]	2021	149	TO	Yes	76.4^b^	33.6	93.6^b^(awake) 46^b^(GA)	0	46.3	5 M	40.9	53	20.1
Das et al*.*, 2022 [2]	2022	46	TO + TS	No	6.27cm^b^	63	*	2.1	32.6	15.2	13	47.8	6.5
Present Series	2023	66	TO	Yes	92.64^b^	39.4	81.2^b^	1.5	44	10.9	50	51.5	22.7

aMedian

bMean

^*^Data not available

Preoperative tumor volume has been shown to be an independent predictor of GTR [7, 11, 16, 25]. Our series had a median tumor volume of 91.9 cc, which was greater than that in most other series, and we found a fairly positive correlation between tumor volume and tumor residue. Larger tumors are more likely to involve multiple zones as well as deeper structures, thus precluding radical resection. Considering the nature of the tumor boundary, we found that radical resection was more technically amenable to sharp tumor borders than to diffuse ones, as is the case in other series [13, 15].

It is well known that insular gliomas follow a very specific growth pattern. Yaşargil & Reeves [35] first reported that limbic gliomas remain confined to the phylogenetic boundaries of the limbic/paralimbic systems in the early stages of growth, and despite their anatomic proximity, IGs usually spare the claustrum, putamen or internal capsule. Here, we grouped the basal forebrain, APS, basal ganglia and internal capsule as deep eloquent regions and found that 78.8% of these tumors involved at least one of these structures, the most common being the basal forebrain and the APS. This region is traditionally considered inviolable, and its involvement negatively affected the EOR according to the univariate analysis.

Ülgen et al. [34] reported that tumors involving the hippocampus have significantly larger postoperative residual tumor volumes than hippocampus-sparing tumors. This makes complete excision of tumors with involvement of the mesial temporal lobe (Yasargil type 5B) unfavorable, as seen in our series. In addition, we also found that contrast-enhancing tumors tend to undergo a greater radical resection rate than non-enhancing tumors; however, it must be borne in mind that when considering EOR, only the contrast-enhancing portion of the postoperative scan was taken as residue, and there may be infiltrative tumors beyond its extent.

The anesthesia protocol (AC versus GA) did not affect the GTR rate according to a systematic review performed by Di Carlo et al*.*[4]; however, Pallud et al*.*[25] and Rossi et al*.*[29] reported that the EOR was significantly greater with awake mapping. In our experience, we did not find such a significant difference. It is likely that giant tumors and the involvement of ‘unresectable regions’, such as Zone II and the APS in many cases, resulted in a reduction in the overall radical resection rates in both groups (awake and GA), and the lower resection rate in the AC could also reflect the fact that, as per our practice, fewer contrast-enhancing tumors (HGGs) underwent surgeries under awake conditions. Non-enhancing lower-grade gliomas are more likely to have residual disease, as was our experience.

### DWI changes and ischemia following insular glioma surgery

Postoperative DWI changes are common after insular glioma surgeries [12]. However, as we have seen, not all these changes translate into clinically relevant infarcts and deficits. Arterial ischemia in particular is important and can lead to long-term deficits, especially those involving the LSA territory. The location of the LSA at the basal forebrain and the APS and its significant impact on postoperative morbidity pose problems during resection.

It has been suggested that LSAs can be preserved by dissecting the MCA proximally until the first perforating arteries are identified and that such dissection defines the deep plane of the tumor [10, 15]. This is possible for type 3A glioma (only 6% of all the tumors in our series), in which complete removal is possible with dissection limited to the base of the superior and inferior periinsular sulci. For all other types, the tumor extends beyond this base through the white matter pathways, and the usual anatomic landmarks are not sufficient for the surgeon to know with reliability the exact location of the perforating arteries on their whole trajectory [5]. Furthermore, intraoperative brain shift reduces the reliability of the identification of operative adjuncts such as neuro-navigation; thus, it is considered safer to leave small residual tumors at depth along the uncinate bundle, especially when the tumor involves the APS [6, 31, 35]. Since the inferior fronto-occipital fasciculus (IFOF) sweeps across the limen insulae just lateral to the APS, stimulation in this region and identification of the IFOF using the pyramid and palm tree test (PPTT) of semantic association can help locate the APS just deep to it and at the same time provide an additional benefit of functionally preserving this important tract.

**Fig. 8:**
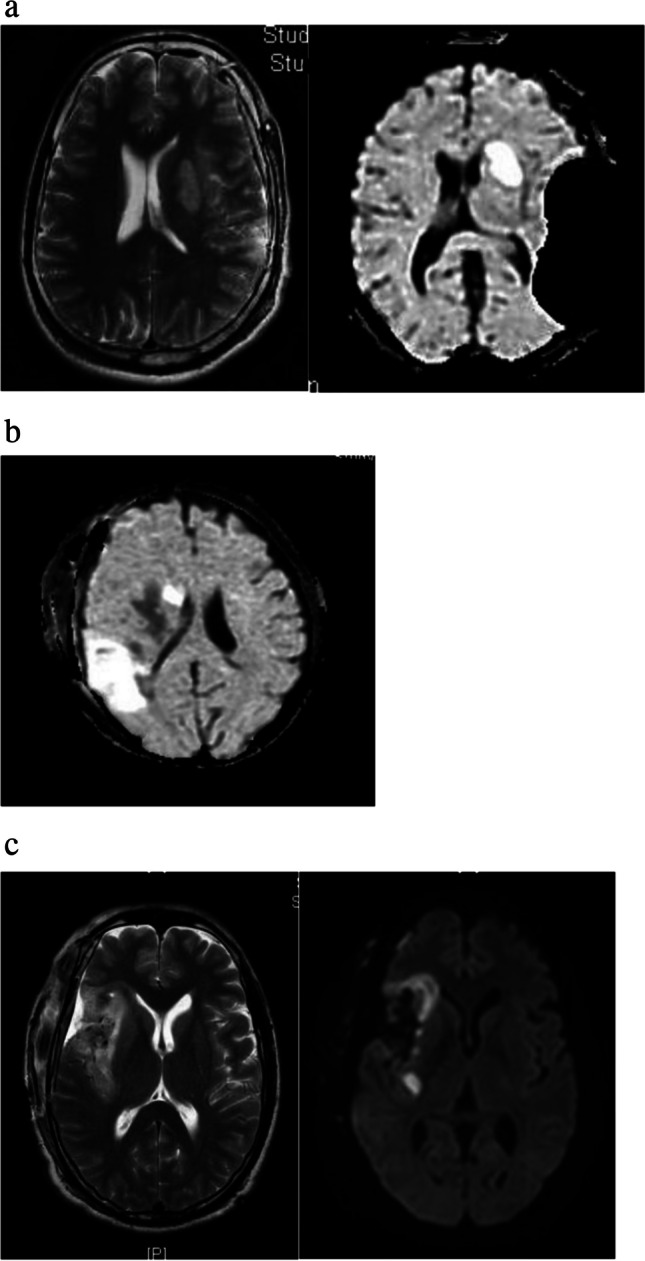
Different types of arterial infarcts on postoperartive MRI- **a** LSA infarct on T2W MRI and DWI, **b** DWI showing a cortical and deep perforator infarct **c** Peri-resection venous infarct on T2W MRI and DWI MRI

Apart from the lateral lenticulostriate vessels, other sources of ischemia are the long insular arteries from the M2-M3 junction and the long medullary arteries emanating from the M4 branches (Fig. 8). The long insular arteries originate from the insular portion of the MCA at the level of the superior insular sulcus and supply the corona radiata and descending motor pathways. The superior extremity of the central insular sulcus (corresponding to zone II of the Berger Sanai classification) can be used as a landmark for long insular artery distribution [13]. In addition, there are other deep perforators from the opercular branches of the M2-M3 segment and the ICA at the anterior choroidal artery origin supplying the posterior limb of the internal capsule, geniculate bodies, optic radiations and globus pallidus, which are implicated in incomplete resection and postoperative deficits [29].

No association between postoperative DWI changes and the EOR was found in our study (SM Table 1). Therefore, one must remember that ischemia can occur at any degree of resection (even with less radical resections), and utmost care should be taken while dissecting throughout IG surgery.

### Neurological deficits

The transient and permanent deficit rates in the literature vary from 14–59% and 0–20%, respectively, as shown in Table 7. In our series, we demonstrated an immediate postoperative deficit rate of 44% and a persistent deficit rate of 10.9%, which is comparable to other series reported in the literature. Various factors were correlated with the risk of deficits. Tumor location is one such factor. Tumors of the dominant hemisphere had a significantly greater rate of immediate postoperative deficits, with 70% (13/21 – language 11, motor 2) of these deficits being transient in nature (*p* = 0.004) and involving the most language. This finding is similar to that reported by Mandonnet [19], where all patients with left-sided insular gliomas had a transient deficit in naming. However, the persistent deficit rates were not significantly different, suggesting the benefit of utilizing AC in many of these patients. This again corroborates the literature regarding stimulation mapping outcomes [3, 4].

Contrary to the literature [9, 11] we did not find an association between B. S zone and morbidity; however, the involvement of the fronto-lateral and parietal operculum was associated with higher immediate deficit rates, and the fronto-orbital operculum was associated with lower prolonged deficit rates. There are 2 main mechanisms of postoperative motor deficit—direct mechanical injury to cortical or subcortical motor substrates by dissection/manipulation or retraction and direct ischemic injury to motor pathways—the latter being more commonly implicated. Although only approximately half (53%) of all patients with postoperative DWI changes had deficits (transient/persistent), it is important to minimize intraoperative ischemia because 20% of those patients who experienced DWI changes developed persistent deficits. The most common ischemic territory was supplied by the LSA (39% of cases of arterial infarcts), which is consistent with the literature [23, 36]. This is also reflected in the higher persistent motor deficit rate associated with ischemia in our series. This emphasizes the importance of exercising utmost care to avoid ischemia during surgery. Various strategies have been suggested. Duffau et al. [5] used intraoperative mapping and identification of IFOF, Das et al. [2] shared their experience using the trans-basisulcal plane as a lateral landmark of the LSA, and Šteňo et al. [33] suggested the use of NUS guided angiography. Conversely, not all patients with deficits had DWI changes, indicating that mechanical injury (without ischemia) can play an important role. Thus, preempting and minimizing neurological deficits during IG resection is of paramount importance. Motor deficits following IG surgery have been associated with a worse prognosis [7], and MEP monitoring helps to safeguard this function intraoperatively. However, this may not always be possible. Apart from its proximity to the pyramidal tract, the anterior insula also harbors connections to the anterior cingulum and dorsal premotor cortex, both of which have been implicated in long-term motor deficits without associated intraoperative MEP changes [8]. Similarly, we found that half of the patients with persistent deficits had stable MEPs, highlighting the limitations of these adjuncts and the need for alternative strategies to preserve motor function on a case-by-case basis. Perhaps by using more sensitive warning criteria, one can pick up these deficits, although, this will increase the risk of false positive warnings. We found that stable Tc MEPs and strip MEPs (with our defined warning criteria) were significantly associated with good motor outcomes, and any change in the amplitude should be regarded cautiously to avoid persistent deficits. Changes in the MEP amplitude constitute the warning criteria, as shown in Table 1, and allow time to take remedial measures. A sudden irreversible loss or deterioration of amplitude suggests a major vascular event (as in one patient in our series). Although reversible deterioration in strip MEPs was associated with DWI changes in 87.5% of the patients (most of whom were arterial), not all of these changes resulted in neurological deficits, suggesting that intraoperative interventions prevented the progression of ischemia to a clinical deficit. Moreover, we were able to achieve radical resection in 62.5% of patients with reversible deterioration in the strip MEP. However, the risk of persistent deficits was greater when reversible MEP changes were associated with DWI changes (than when no DWI changes were observed). This could suggest ischemic injury at surgery, which progressed postoperatively despite the MEP change being reversible. This could again argue for the use of more stringent warning criteria for reversible MEP changes. The irreversible loss of MEPs, on the other hand, almost certainly predicted a major ischemic event and precluded further resection. This finding suggests the role of MEP monitoring in recognizing the presence of ischemia, taking appropriate measures to prevent persistent motor deficits, guiding EOR, and identifying patients with poor motor prognoses who will require aggressive rehabilitation.

According to a meta-analysis by Di Carlo et al. [4], AC is associated with greater immediate deficit rates and lower permanent deficit rates than GA. Although we did find greater transient and persistent deficits in the AC group, the difference was not significant (10% for the GA group and 15% for the awake group for persistent deficit rates). The absence of this difference does not necessarily mean that GA is comparable to AC because of the diverse nature of the cohorts. AC was performed more often for tumors of the dominant lobe, resulting in a persistent deficit rate that was not significantly different from that in the nondominant lobe, as seen in the multivariate analysis. Perhaps a study with a larger sample size would further elucidate this effect.

## Limitations

Our study has several limitations. First, this study has the usual constraints of a retrospective design. Second, the size of the cohort was small and heterogeneous. Third, this study was conducted at a single institution, and since surgical outcomes are highly dependent on surgical skill, the results cannot be compared with those of other studies. However, the detailed analysis of outcomes and associated factors provides invaluable insights and practical lessons that can be implemented during TO surgery for insular gliomas.

## Conclusion

Radical resection of IGs is possible in many cases, although gross total resection is much more difficult, especially for giant lower-grade infiltrative tumors involving Zone II. Ischemic injury is a major cause of deficits. The use of MEPs is helpful for identifying and preventing irreversible motor deficits; however, some deficits may arise even with stable MEPs. AC is helpful for treating dominant lobe tumors. Ischemic insults, especially in the LSA territory, can be extremely damaging. Meticulous dissection should be employed along with judicious use of intraoperative adjuncts to minimize morbidity.

## Supplementary Information

Below is the link to the electronic supplementary material.

Supplementary file1 (DOCX 64.7 KB)

## Data Availability

All data are available with the authors and will be made available upon reasonable request.

## Abbreviations

IGs: Insular Gliomas
TO: Transopercular
MEPs: Motor Evoked Potentials
Tc MEPS: Transcranial Motor Evoked Potentials
DWI: Diffusion Weighted Imaging
IONM: Intra-Operative Neuro-Monitoring
TS: Trans-sylvian
EMR: Electronic Medical Records
PACS: Picture Archiving and Communication System
AC: Awake Craniotomy
GA: General Anesthesia
DCS: Direct Cortical Stimulation
EOR: Extent of Resection
MCA: Middle Cerebral Artery
LSA: Lenticulostriate Artery
IDH: Isocitrate Dehydrogenase
APS: Anterior Perforated Substance
5’ALA: 5’ Amino Levulinic Acid
GTR: Gross Total Resection
HGG: High Grade Glioma
IFOF: Inferior Fronto-occipital Fasciculus
PPTT: Pyramid and Palm tree test
B.S zone: Berger Sanai Zone
NUS: Navigation Guided Ultrasound
