# Supplementary material for: Factors affecting the extent of resection and neurological outcomes following transopercular resection of insular gliomas
Source: Acta Neurochir (Wien). 2024 Jun 1;166(1):244. doi: 10.1007/s00701-024-06124-9 (PMC11144144; doi:10.1007/s00701-024-06124-9)
Supplement: Supplementary file 1 — Supplementary file1 (DOCX 64.7 KB) [file 701_2024_6124_MOESM1_ESM.docx]

Supplementary Table 1. Infarct type and percentages and its correlation with Extent of resection.

| **Infarct type** | **Number of patients (%)(N=66)** | **>90% EOR (N= 26)**  **n(%)** | **<90% EOR (N= 40)**  **n(%)** | **P value** |
| --- | --- | --- | --- | --- |
| Arterial Infarct | 17(25.7) | 4(15.4) | 13(32.5) | 0.10 |
| Venous (Thick peri-resection rim) | 14(34.1) | 7(26.9) | 7(17.5) | 0.27 |
| Both arterial and venous infarct | 1(1.5) | 11(42.3) | 19(47.5) | 0.43 |

Supplementary Table 2. Effect of resection under awake/GA on Type of deficit:

|  | | Awake resection (N=33) n(%) | Resection under GA (N=33) n(%) | P value |
| --- | --- | --- | --- | --- |
| Dominant side tumors | | 21(63.6%) | 13(39.4%) | **0.04** |
| Motor Deficit | Transient motor deficit | 1(3%) | 7(21.2%) | **0.05** |
|  | Persistent motor deficit | 3(9.1%)* | 3(9.1%) | 1 |
| Speech Deficit | Transient speech deficit | 7(21.2%) | 4(12.1%) | 0.51 |
|  | Persistent speech deficit | 2(6.1%) | 1(3%) | 1 |

*1 of the deficits was a delayed motor deficit, due to operative bed hematoma requiring evacuation.

Supplementary Table 3. Analysis of factors affecting EOR.

| Variable Name | Category | Univariate | | | Multivariate | | | |
| --- | --- | --- | --- | --- | --- | --- | --- | --- |
|  |  | EOR | | p-value | Odds Ratio | 95.0% CI for OR | | p-value |
|  |  | >90% Resection | <90% Resection |  |  | Lower | Upper |  |
| Prior Treatment | No | 21(80.8) | 31(77.5) | 0.501 |  |  |  |  |
|  | Yes | 5(19.2) | 9(22.5) |  |  |  |  |  |
| Type of Prior Treatment | Surgery | 4(80.2) | 6(66.7) | 0.504 |  |  |  |  |
|  | Surgery+RT | 1(20) | 1(11.1) |  |  |  |  |  |
|  | Surgery+RT+CT | 0 | 2(22.2) |  |  |  |  |  |
| Histological Class | Astrocytic | 15(57.7) | 22(55) | 0.920 |  |  |  |  |
|  | GBM | 5(19.2) | 7(17.5) |  |  |  |  |  |
|  | Oligo | 6(23.1) | 11(27.5) |  |  |  |  |  |
| Side Of SOL | Left | 13(50) | 21(52.5) | 0.521 |  |  |  |  |
|  | Right | 13(50) | 19(47.5) |  |  |  |  |  |
| Awake Craniotomy | No | 14(53.8) | 19(47.5) | 0.401 |  |  |  |  |
|  | Yes | 12(46.2) | 21(52.5) |  |  |  |  |  |
| Neuromonitoring Done | No | 1(3.8) | 2(5) | 0.659 |  |  |  |  |
|  | Yes | 25(96.2) | 38(95) |  |  |  |  |  |
| Tc MEP done | No | 11(42.3) | 22(55) | 0.225 |  |  |  |  |
|  | Yes | 15(57.7) | 18(45) |  |  |  |  |  |
| Strip MEP done | No | 6(23.1) | 4(10) | 0.137 |  |  |  |  |
|  | Yes | 20(76.9) | 36(90) |  |  |  |  |  |
| Subcortical Mapping done | No | 15(57.7) | 21(52.5) | 0.437 |  |  |  |  |
|  | Yes | 11(42.3) | 19(47.5) |  |  |  |  |  |
| US used | No | 0 | 2(5) | 0.364 |  |  |  |  |
|  | Yes | 26(100) | 38(95) |  |  |  |  |  |
| ALA used | No | 22(84.6) | 40(100) | 0.021 |  |  |  |  |
|  | Yes | 4(15.4) | 0 |  |  |  |  |  |
| Navigation used | No | 3(11.5) | 11(27.5) | 0.105 |  |  |  |  |
|  | Yes | 23(88.5) | 29(72.5) |  |  |  |  |  |
| Deep Regions | No | 10(38.5) | 4(10) | 0.007 |  |  |  |  |
|  | Yes | 16(61.5) | 36(90) |  |  |  |  |  |
| Anterior Insula | No | 6(23.1) | 8(20) | 0.499 |  |  |  |  |
|  | Yes | 20(76.9) | 32(80) |  |  |  |  |  |
| Posterior Insula | No | 5(19.2) | 3(7.5) | 0.149 |  |  |  |  |
|  | Yes | 21(80.8) | 37(92.5) |  |  |  |  |  |
| Fronto-orbital operculum | No | 15(57.7) | 15(37.5) | 0.087 |  |  |  |  |
|  | Yes | 11(42.3) | 25(62.5) |  |  |  |  |  |
| Fronto-lateral operculum | No | 14(53.8) | 15(37.5) | 0.146 |  |  |  |  |
|  | Yes | 12(46.2) | 25(62.5) |  |  |  |  |  |
| Parietal operculum | No | 20(76.9) | 27(67.5) | 0.294 |  |  |  |  |
|  | Yes | 6(23.1) | 13(32.5) |  |  |  |  |  |
| Mesial Temporal Lobe | No | 16(61.5) | 12(30) | 0.011 |  |  |  |  |
|  | Yes | 10(38.5) | 28(70) |  |  |  |  |  |
| Lateral Temporal Lobe | No | 13(50) | 17(42.5) | 0.365 |  |  |  |  |
|  | Yes | 13(50) | 23(57.5) |  |  |  |  |  |
| Zone I Berger Sanai | No | 12(46.2) | 11(27.5) | 0.099 |  |  |  |  |
|  | Yes | 14(53.8) | 29(72.5) |  |  |  |  |  |
| Zone II Berger Sanai | No | 14(53.8) | 10(25) | 0.017 |  |  |  |  |
|  | Yes | 12(46.2) | 30(75) |  | 0.282 | 0.091 | 0.875 | 0.028 |
| Zone III Berger Sanai | No | 9(34.6) | 9(22.5) | 0.212 |  |  |  |  |
|  | Yes | 17(65.4) | 31(77.5) |  |  |  |  |  |
| Zone IV Berger Sanai | No | 7(26.9) | 10(25) | 0.541 |  |  |  |  |
|  | Yes | 19(73.1) | 30(75) |  |  |  |  |  |
| Number of Berger Sanai Zones | 1 | 5(19.2) | 1(9.1) | 0.053 |  |  |  |  |
|  | 2 | 12(46.2) | 17(42.5) |  |  |  |  |  |
|  | 3 | 3(11.5) | 3(7.5) |  |  |  |  |  |
|  | 4 | 6(23.1) | 19(47.5) |  |  |  |  |  |
| Yasargil Type | 3A | 2(7.7) | 2(5) | 0.089 |  |  |  |  |
|  | 3B | 4(15.4) | 3(7.5) |  | 3.956 | 0.329 | 47.572 | 0.278 |
|  | 5A | 10(38.5) | 7(17.5) |  | 2.488 | 0.290 | 21.347 | 0.406 |
|  | 5B | 10(38.5) | 28(70) |  | 0.732 | 0.096 | 5.569 | 0.763 |
| Localization | Diffuse | 3(11.5) | 16(40) | 0.044 |  |  |  |  |
|  | Moderately Localised | 15(57.7) | 15(37.5) |  |  |  |  |  |
|  | Well Localised | 8(30.8) | 9(22.5) |  |  |  |  |  |
| Consistency | Pred Solid | 20(76.9) | 28(70) | 0.102 |  |  |  |  |
|  | Pred Cystic | 4(15.4) | 2(5) |  |  |  |  |  |
|  | Mixed | 2(7.7) | 10(25) |  |  |  |  |  |
| Enhancement | Non Enhancing | 8(30.8) | 23(57.5) | 0.030 |  |  |  |  |
|  | Enhancing | 18(69.2) | 17(42.5) |  | 3.023 | 1.005 | 9.090 | 0.049 |

Supplementary Table 4. Analysis of factors affecting Neurological Outcomes.

| **Variable Name** | Category | Univariate | | | Multivariate | | | | Univariate | | | Multivariate | | | | Univariate | | | Multivariate | | | |
| --- | --- | --- | --- | --- | --- | --- | --- | --- | --- | --- | --- | --- | --- | --- | --- | --- | --- | --- | --- | --- | --- | --- |
|  |  | **Immediate Deficit** | | p-value | Odds Ratio | 95.0% CI for OR | | p-value | **Prolonged Deficit** | | p-value | Odds Ratio | 95.0% CI for OR | | p-value | **Persistent Deficit** | | p-value | Odds Ratio | 95.0% CI for OR | | p-value |
|  |  | No | Yes |  |  | Lower | Upper |  | No | Yes |  |  | Lower | Upper |  | No | Yes |  |  | Lower | Upper |  |
| **Prior**  **Treatment** | No | 27(75%) | 25(83.3%) | 0.55 |  |  |  |  | 40(76.9%) | 12(85.7%) | 0.72 |  |  |  |  | 42(76.4%) | 8(100%) | 0.19 | 0.79 | 0.11 | 5.41 | 0.81 |
|  | Yes | 9(25%) | 5(16.7%) |  |  |  |  |  | 12(23.1%) | 2(14.3%) |  |  |  |  |  | 13(23.6%) | 0(0%) |  |  |  |  |  |
| **Type of**  **Prior Treatment** | Surgery | 5(55.6%) | 5(100%) | 0.21 |  |  |  |  | 8(66.7%) | 2(100%) | 0.63 |  |  |  |  | 9(69.2%) | -- |  |  |  |  |  |
|  | Surgery  +RT | 2(22.2%) | 0(0%) |  |  |  |  |  | 2(16.7%) | 0(0%) |  |  |  |  |  | 2(15.4%) | -- |  |  |  |  |  |
|  | Surgery  +RT+CT | 2(22.2%) | 0(0%) |  |  |  |  |  | 2(16.7%) | 0(0%) |  |  |  |  |  | 2(15.4%) | -- |  |  |  |  |  |
| **Histological**  **Class** | Astrocytic | 20(55.6%) | 17(56.7%) | 0.58 |  |  |  |  | 27(51.9%) | 10(71.4%) | 0.35 |  |  |  |  | 29(52.7%) | 5(62.5%) | 0.84 |  |  |  |  |
|  | GBM | 8(22.2%) | 4(13.3%) |  |  |  |  |  | 11(21.2%) | 1(7.1%) |  |  |  |  |  | 11(20%) | 1(12.5%) |  |  |  |  |  |
|  | Oligo | 8(22.2%) | 9(30%) |  |  |  |  |  | 14(26.9%) | 3(21.4%) |  |  |  |  |  | 15(27.3%) | 2(25%) |  |  |  |  |  |
| **Side Of SOL** | Left | 13(36.1%) | 21(70%) | 0.01 |  |  |  |  | 25(48.1%) | 9(64.3%) | 0.37 |  |  |  |  | 26(47.3%) | 7(87.5%) | 0.06 |  |  |  |  |
|  | Right | 23(63.9%) | 9(30%) |  | 0.26 | 0.08 | 0.78 | 0.02 | 27(51.9%) | 5(35.7%) |  |  |  |  |  | 29(52.7%) | 1(12.5%) |  | 0.36 | 0.09 | 1.46 | 0.15 |
| **Awake**  **Craniotomy** | No | 17(47.2%) | 16(53.3%) | 0.80 |  |  |  |  | 26(50%) | 7(50%) | 1.00 |  |  |  |  | 27(49.1%) | 3(37.5%) | 0.71 |  |  |  |  |
|  | Yes | 19(52.8%) | 14(46.7%) |  |  |  |  |  | 26(50%) | 7(50%) |  |  |  |  |  | 28(50.9%) | 5(62.5%) |  |  |  |  |  |
| **Neuromonitoring**  **Done** | No | 2(5.6%) | 1(3.3%) | 1.00 |  |  |  |  | 3(5.8%) | 0(0%) | 1.00 |  |  |  |  | 3(5.5%) | 0(0%) | 1.00 |  |  |  |  |
|  | Yes | 34(94.4%) | 29(96.7%) |  |  |  |  |  | 49(94.2%) | 14(100%) |  |  |  |  |  | 52(94.5%) | 8(100%) |  |  |  |  |  |
| **Tc MEP done** | No | 19(52.8%) | 14(46.7%) | 0.80 |  |  |  |  | 26(50%) | 7(50%) | 1.00 |  |  |  |  | 28(50.9%) | 5(62.5%) | 0.71 |  |  |  |  |
|  | Yes | 17(47.2%) | 16(53.3%) |  |  |  |  |  | 26(50%) | 7(50%) |  |  |  |  |  | 27(49.1%) | 3(37.5%) |  |  |  |  |  |
| **Strip MEP done** | No | 7(19.4%) | 3(10%) | 0.33 |  |  |  |  | 9(17.3%) | 1(7.1%) | 0.67 |  |  |  |  | 10(18.2%) | 0(0%) | 0.33 |  |  |  |  |
|  | Yes | 29(80.6%) | 27(90%) |  |  |  |  |  | 43(82.7%) | 13(92.9%) |  |  |  |  |  | 45(81.8%) | 8(100%) |  |  |  |  |  |
| **Subcortical**  **Mapping done** | No | 22(61.1%) | 14(46.7%) | 0.32 |  |  |  |  | 30(57.7%) | 6(42.9%) | 0.37 |  |  |  |  | 32(58.2%) | 4(50%) | 0.71 |  |  |  |  |
|  | Yes | 14(38.9%) | 16(53.3%) |  |  |  |  |  | 22(42.3%) | 8(57.1%) |  |  |  |  |  | 23(41.8%) | 4(50%) |  |  |  |  |  |
| **US used** | No | 1(2.8%) | 1(3.3%) | 1.00 |  |  |  |  | 1(1.9%) | 1(7.1%) | 0.38 |  |  |  |  | 2(3.6%) | 0(0%) | 1.00 |  |  |  |  |
|  | Yes | 35(97.2%) | 29(96.7%) |  |  |  |  |  | 51(98.1%) | 13(92.9%) |  |  |  |  |  | 53(96.4%) | 8(100%) |  |  |  |  |  |
| **ALA used** | No | 35(97.2%) | 27(90%) | 0.32 |  |  |  |  | 48(92.3%) | 14(100%) | 0.57 |  |  |  |  | 51(92.7%) | 8(100%) | 1.00 |  |  |  |  |
|  | Yes | 1(2.8%) | 3(10%) |  |  |  |  |  | 4(7.7%) | 0(0%) |  |  |  |  |  | 4(7.3%) | 0(0%) |  |  |  |  |  |
| **Navigation used** | No | 10(27.8%) | 4(13.3%) | 0.23 |  |  |  |  | 13(25%) | 1(7.1%) | 0.27 |  |  |  |  | 14(25.5%) | 0(0%) | 0.18 |  |  |  |  |
|  | Yes | 26(72.2%) | 26(86.7%) |  |  |  |  |  | 39(75%) | 13(92.9%) |  |  |  |  |  | 41(74.5%) | 8(100%) |  | 8.30 | 0.45 | 153.06 | 0.15 |
| **Deep Regions** | No | 7(19.4%) | 7(23.3%) | 0.77 |  |  |  |  | 11(21.2%) | 3(21.4%) | 1.00 |  |  |  |  | 13(23.6%) | 1(12.5%) | 0.67 |  |  |  |  |
|  | Yes | 29(80.6%) | 23(76.7%) |  |  |  |  |  | 41(78.8%) | 11(78.6%) |  |  |  |  |  | 42(76.4%) | 7(87.5%) |  |  |  |  |  |
| **Anterior Insula** | No | 7(19.4%) | 7(23.3%) | 0.77 |  |  |  |  | 10(19.2%) | 4(28.6%) | 0.47 |  |  |  |  | 12(21.8%) | 2(25%) | 1.00 |  |  |  |  |
|  | Yes | 29(80.6%) | 23(76.7%) |  |  |  |  |  | 42(80.8%) | 10(71.4%) |  |  |  |  |  | 43(78.2%) | 6(75%) |  |  |  |  |  |
| **Posterior Insula** | No | 5(13.9%) | 3(10%) | 0.72 |  |  |  |  | 7(13.5%) | 1(7.1%) | 1.00 |  |  |  |  | 7(12.7%) | 1(12.5%) | 1.00 |  |  |  |  |
|  | Yes | 31(86.1%) | 27(90%) |  |  |  |  |  | 45(86.5%) | 13(92.9%) |  |  |  |  |  | 48(87.3%) | 7(87.5%) |  |  |  |  |  |
| **Fronto-orbital**  **operculum** | No | 15(41.7%) | 15(50%) | 0.62 |  |  |  |  | 21(40.4%) | 9(64.3%) | 0.14 |  |  |  |  | 25(45.5%) | 3(37.5%) | 0.72 |  |  |  |  |
|  | Yes | 21(58.3%) | 15(50%) |  |  |  |  |  | 31(59.6%) | 5(35.7%) |  | 0.25 | 0.06 | 0.99 | 0.05 | 30(54.5%) | 5(62.5%) |  |  |  |  |  |
| **Fronto-lateral**  **operculum** | No | 20(55.6%) | 9(30%) | 0.05 |  |  |  |  | 24(46.2%) | 5(35.7%) | 0.56 |  |  |  |  | 26(47.3%) | 3(37.5%) | 0.72 |  |  |  |  |
|  | Yes | 16(44.4%) | 21(70%) |  | 1.81 | 0.52 | 6.3 | 0.35 | 28(53.8%) | 9(64.3%) |  |  |  |  |  | 29(52.7%) | 5(62.5%) |  |  |  |  |  |
| **Parietal operculum** | No | 30(83.3%) | 17(56.7%) | 0.03 |  |  |  |  | 40(76.9%) | 7(50%) | 0.09 |  |  |  |  | 41(74.5%) | 4(50%) | 0.21 |  |  |  |  |
|  | Yes | 6(16.7%) | 13(43.3%) |  | 1.72 | 0.45 | 6.58 | 0.43 | 12(23.1%) | 7(50%) |  | 2.05 | 0.53 | 8.00 | 0.30 | 14(25.5%) | 4(50%) |  |  |  |  |  |
| **Mesial Temporal**  **Lobe** | No | 12(33.3%) | 16(53.3%) | 0.13 |  |  |  |  | 22(42.3%) | 6(42.9%) | 1.00 |  |  |  |  | 23(41.8%) | 3(37.5%) | 1.00 |  |  |  |  |
|  | Yes | 24(66.7%) | 14(46.7%) |  | 0.52 | 0.16 | 1.65 | 0.26 | 30(57.7%) | 8(57.1%) |  |  |  |  |  | 32(58.2%) | 5(62.5%) |  |  |  |  |  |
| **Lateral Temporal**  **Lobe** | No | 13(36.1%) | 17(56.7%) | 0.14 |  |  |  |  | 25(48.1%) | 5(35.7%) | 0.55 |  |  |  |  | 26(47.3%) | 3(37.5%) | 0.72 |  |  |  |  |
|  | Yes | 23(63.9%) | 13(43.3%) |  | 0.48 | 0.15 | 1.49 | 0.20 | 27(51.9%) | 9(64.3%) |  |  |  |  |  | 29(52.7%) | 5(62.5%) |  |  |  |  |  |
| **Berger Sanai**  **Zone I** | No | 13(36.1%) | 10(33.3%) | 1.00 |  |  |  |  | 18(34.6%) | 5(35.7%) | 1.00 |  |  |  |  | 21(38.2%) | 2(25%) | 0.7 |  |  |  |  |
|  | Yes | 23(63.9%) | 20(66.7%) |  |  |  |  |  | 34(65.4%) | 9(64.3%) |  |  |  |  |  | 34(61.8%) | 6(75%) |  |  |  |  |  |
| **Zone II** | No | 15(41.7%) | 9(30%) | 0.44 |  |  |  |  | 20(38.5%) | 4(28.6%) | 0.55 |  |  |  |  | 21(38.2%) | 2(25%) | 0.7 |  |  |  |  |
|  | Yes | 21(58.3%) | 21(70%) |  |  |  |  |  | 32(61.5%) | 10(71.4%) |  |  |  |  |  | 34(61.8%) | 6(75%) |  |  |  |  |  |
| **Zone III** | No | 8(22.2%) | 10(33.3%) | 0.41 |  |  |  |  | 13(25%) | 5(35.7%) | 0.50 |  |  |  |  | 14(25.5%) | 2(25%) | 1.00 |  |  |  |  |
|  | Yes | 28(77.8%) | 20(66.7%) |  |  |  |  |  | 39(75%) | 9(64.3%) |  |  |  |  |  | 41(74.5%) | 6(75%) |  |  |  |  |  |
| **Zone IV** | No | 7(19.4%) | 10(33.3%) | 0.26 |  |  |  |  | 13(25%) | 4(28.6%) | 0.74 |  |  |  |  | 14(25.5%) | 2(25%) | 1.00 |  |  |  |  |
|  | Yes | 29(80.6%) | 20(66.7%) |  |  |  |  |  | 39(75%) | 10(71.4%) |  |  |  |  |  | 41(74.5%) | 6(75%) |  |  |  |  |  |
| **Number of**  **Berger Sanai Zones** | 1 | 1(2.8%) | 5(16.7%) | 0.21 |  |  |  |  | 5(9.6%) | 1(7.1%) | 0.96 |  |  |  |  | 6(10.9%) | 0(0%) | 0.53 |  |  |  |  |
|  | 2 | 18(50%) | 11(36.7%) |  |  |  |  |  | 22(42.3%) | 7(50%) |  |  |  |  |  | 23(41.8%) | 4(50%) |  |  |  |  |  |
|  | 3 | 4(11.1%) | 2(6.7%) |  |  |  |  |  | 5(9.6%) | 1(7.1%) |  |  |  |  |  | 6(10.9%) | 0(0%) |  |  |  |  |  |
|  | 4 | 13(36.1%) | 12(40%) |  |  |  |  |  | 20(38.5%) | 5(35.7%) |  |  |  |  |  | 20(36.4%) | 4(50%) |  |  |  |  |  |
| **Yasargil Type** | 3A | 2(5.6%) | 2(6.7%) | 0.33 |  |  |  |  | 4(7.7%) | 0(0%) | 0.06 |  |  |  |  | 4(7.3%) | 0(0%) | 0.32 |  |  |  |  |
|  | 3B | 2(5.6%) | 5(16.7%) |  |  |  |  |  | 3(5.8%) | 4(28.6%) |  | 3.38 | 0.10 | 113.05 | 0.5 | 4(7.3%) | 2(25%) |  |  |  |  |  |
|  | 5A | 8(22.2%) | 9(30%) |  |  |  |  |  | 15(28.8%) | 2(14.3%) |  | 0.94 | 0.03 | 26.74 | 0.97 | 15(27.3%) | 1(12.5%) |  |  |  |  |  |
|  | 5B | 24(66.7%) | 14(46.7%) |  |  |  |  |  | 30(57.7%) | 8(57.1%) |  | 1.65 | 0.07 | 39.18 | 0.76 | 32(58.2%) | 5(62.5%) |  |  |  |  |  |
| **Localization** | Diffuse | 12(33.3%) | 7(23.3%) | 0.40 |  |  |  |  | 15(28.8%) | 4(28.6%) | 0.96 |  |  |  |  | 16(29.1%) | 3(37.5%) | 0.88 |  |  |  |  |
|  | Moderately  Localised | 17(47.2%) | 13(43.3%) |  |  |  |  |  | 24(46.2%) | 6(42.9%) |  |  |  |  |  | 25(45.5%) | 3(37.5%) |  |  |  |  |  |
|  | Well  Localised | 7(19.4%) | 10(33.3%) |  |  |  |  |  | 13(25%) | 4(28.6%) |  |  |  |  |  | 14(25.5%) | 2(25%) |  |  |  |  |  |
| **Consistency** | Pred Solid | 24(66.7%) | 24(80%) | 0.49 |  |  |  |  | 35(67.3%) | 13(92.9%) | 0.15 |  |  |  |  | 38(69.1%) | 7(87.5%) | 0.49 |  |  |  |  |
|  | Pred Cystic | 4(11.1%) | 2(6.7%) |  |  |  |  |  | 6(11.5%) | 0(0%) |  | 0.39 | 0.02 | 8.99 | 0.56 | 6(10.9%) | 0(0%) |  |  |  |  |  |
|  | Mixed | 8(22.2%) | 4(13.3%) |  |  |  |  |  | 11(21.2%) | 1(7.1%) |  | 0.43 | 0.06 | 3.24 | 0.41 | 11(20%) | 1(12.5%) |  |  |  |  |  |
| **Enhancement** | Non  Enhancing | 15(41.7%) | 16(53.3%) | 0.46 |  |  |  |  | 21(40.4%) | 10(71.4%) | 0.07 |  |  |  |  | 24(43.6%) | 5(62.5%) | 0.45 |  |  |  |  |
|  | Enhancing | 21(58.3%) | 14(46.7%) |  |  |  |  |  | 31(59.6%) | 4(28.6%) |  | 0.37 | 0.09 | 1.56 | 0.17 | 31(56.4%) | 3(37.5%) |  |  |  |  |  |
